# Small-Scale Farmers Associations’ adaptive capacity to climate change in Swaziland sugarcane industry

**DOI:** 10.4102/jamba.v11i2.697

**Published:** 2019-06-25

**Authors:** Bon’sile F.N. Mhlanga-Ndlovu, Godwell Nhamo

**Affiliations:** 1 Department of Environmental Sciences, University of South Africa, Johannesburg, South Africa; 2 Institute for Corporate Citizenship, University of South Africa, Johannesburg, South Africa

**Keywords:** Swaziland, Sugar, Adaptation, Climate Change, Farmers

## Abstract

This study investigated the existing adaptive capacity for climate change impacts by Small-Scale Famers Associations (SSFAs) in Swaziland’s sugar industry. The analysis of adaptive capacity considered how the livelihood assets (natural, physical, financial, human and social) as discussed in the Sustainable Livelihoods Framework (SLF) help promote SSFAs’ adaptive capacity to climate change. The study took place in the Lowveld. Data were generated through a questionnaire from 45 SSFAs supervisors representing more than 2700 farmers. In addition, face-to-face interviews were undertaken with key informants, namely, Swaziland Water and Agricultural Development Enterprise, Swaziland Sugar Association, Ministry of Natural Resources and Energy, Ministry of Agriculture, Ministry of Economic Planning and Development, and the United Nations Development Programme. The results indicate that the farmers have less adaptive capacity, and this affects the implementation of adaptation measures. The priority action towards increased adaptation includes interventions on credit, utility costs and taxes, land resources ownership and management, as well as information dissemination, especially early warning.

## Introduction

Climate change has become the primary environmental concern of the 21st century (Dessler & Parson 2006). According to the Intergovernmental Panel on Climate Change (IPCC), Africa remains one of the most vulnerable continents to climate change because of its poor adaptive capacity (IPCC 2014). Among the representative key risks for the region are compounded stress on water resources, reduced crop productivity and livelihood as well as food insecurity. Researchers have observed temperature increases coupled with impacts on the physical and biological systems over the past century (Maponya & Sylvester 2012). Literature indicates that any future change in climate will most certainly have some form of impact, not only on conditions of the physical environment but also on the overall socio-economic aspects of life. Climate change effects on rainfall and temperature render agriculture the most susceptible sector (Mahendra 2011).

Swaziland faces a major decline in the Gross Domestic Product (GDP) as a result of climate change impacts. This is because the main economic growth and development contributing sectors such as agriculture and water resources are also climate sensitive (SSA 2013). The Swaziland Sugar Association (SSA) maintains that the sugar industry provides 59% of agricultural output, 35% of agricultural wage employment and about 8% to the country’s GDP output (SSA 2011). The projected climate change impacts resulting in reduced water availability, health stress on the labour force and natural resources degradation will, therefore, affect the sector’s contribution towards GDP (Deressa, Hassan & Poonyth 2005). The major issue, therefore, is whether Small-Scale Farmers Associations (SSFAs) in the Lowveld sugar industry have the required adaptive capacity to climate change.

This study seeks to determine climate change adaptive capacities associated with SSFAs involved in sugarcane farming in Swaziland. The adaptive capacity assessment followed the Sustainable Livelihoods Framework (SLF) as presented by the Department for International Development – DFID (DFID 1999). The SLF assists in assessing access to and ownership of natural, financial, human, physical and social capital assets necessary to cope with climate variability and change. To this end, a single research question and objective were established. What adaptation measures are SSFAs in the sugarcane industry of Swaziland’s Lowveld region employing in response to the changing climate and what are the barriers to climate change adaptation? The objective was to assess adaptive capacities of these SSFAs within the context of the SLF.

## Literature review

Adaptive capacity is considered by Engle (2011) as the positive features of a system to reduce vulnerability. Adaptive capacity represents the set of both biophysical and socio-economic factors that determine people’s ability to cope with stress or change in terms of the likelihood of occurrence and impacts of weather and climate-related events (Nicholls, Hoozemans & Marchand 1999). Research indicates that farmers’ ability to perceive climate change is a very critical precondition for their resultant choice to adapt (Gbetibouo 2009). Decentralising power to local levels such as SSFAs to promote adaptive capacity against climate change stresses will have to include planning, management and monitoring of natural and other resources (WRI 2009).

Deressa et al. (2005) undertook a study on climate change impact on sugarcane by means of the Ricardian Model for both rain-fed and irrigated production. The results indicated that sugarcane production is highly vulnerable to changes in climate conditions. Another study conducted on climate change impact on sugarcane by the Southern African Sugar Industry (SASA) revealed that the total revenue per hectare of sugarcane is likely to decrease with projected temperature intensification (SASA 2006). The report further stated that changes in precipitation would also impact sugarcane production, but not as severely as temperature changes would. The studies show that less land would be used for the cultivation of sugarcane because of the changes in climate that renders land and soil no longer suitable for sugarcane cultivation (SASA 2006). In an earlier study, the SASA reveals that there would be an increase in flash floods and a tendency towards a warmer climate (SASA 2002). With such trends, insects, pests and diseases were expected to thrive as the ecological balance gets disrupted. This viewpoint is confirmed by Nayamuth and Nayamuth (2002) who maintain that insects may colonise new areas and new species could move into sugarcane growing areas. Land suitability may change, resulting in a shift in sugarcane growing areas, thus competing with other crops for appropriate arable land and growing areas. Land-use changes will have to be analysed in relation to mills and infrastructure as well as the surrounding communities (Dale 1997). A deterioration of sugarcane quality will potentially reduce milling efficiencies, and the cost of production and increase in sugar prices in the world market could reduce the adaptive capacity of farmers such as SSFAs (Parry et al. 2004).

The issue of sucrose decrease is also of concern to climate change and adaptive capacities of SSFAs. A decrease in sucrose yield will need to be countered by mass irrigation, the growing of drought-resistant varieties and a change in crop cycles, while the negative effects of climate change may be countered by a rise in atmospheric carbon dioxide, which is essential for plant growth (SASA 2002). All these factors point to an increased vulnerability, necessitating improved adaptability if the sugarcane industry, including the SSFAs in Swaziland, has to thrive.

The documented environmental, economic and social impacts of climate change (Schulze & Dlamini 2005) indicate some negative consequences for the agriculture sector in Swaziland. Predicted climate change impacts include a reduction of rainfall, increased evaporation rates, decreased run-off and aggravation of droughts, salinisation, wind and dust storms (because of landscape degradation and land clearing) and increased frequency of extreme climatic events (SASA 2002). On studying three catchments in Swaziland, namely, Komati, Mbuluzi and Ngwavuma (Matondo, Peter & Msibi 2004), the authors concluded that stream flow in these catchments will be reduced under changing climate conditions. The expected decreases in stream flow because of climate change will necessitate the implementation of policies and strategies that will promote practices for conserving water resources (Matondo, Peter & Msibi 2004). A study on the Mbuluzi catchment, which feeds the Mnjoli Dam (a reservoir used primarily for storing irrigation water for sugarcane within the Royal Swaziland Sugar Corporation [RSSC]), concluded that with a 2°C increase in temperature, coupled with a 10% reduction in precipitation, inflows to the reservoir will reduce by about 34% in median years (Schulze & Dlamini 2005). This condition indicates the level of vulnerability the sugarcane industry will face as a result of climate change impacting on the availability of water resources and at the right time for irrigation purposes.

A study on change impacts and adaptation of the Swaziland sugar industry by Knox et al. (2010) indicate that climate change is already affecting sugarcane farmers in the country. The authors indicate that climate change will likely render the current peak capacity of existing sugarcane irrigation schemes in Swaziland inadequate to fulfil the projected increases in irrigation demand in almost 50% of the years under unlimited water availability. Climate change will place more pressure on the existing challenges that include loss of farm productivity and change in land suitability for current agriculture commodities (Tsabedze 2005).

Water resources in Swaziland are predicted to become increasingly limited because of climate change (World Bank 2007). To this end, technologies that combine soil fertility improvement and the storage and the efficient use of water will become essential to ensure adaptation within the agricultural systems (Ahmed, Sanders & Nell 2000). Soil and water conservation is considered beneficial because this has the potential to (1) improve soil fertility, (2) enhance soil water storage and (3) ensure crop buffer against droughts and floods (World Bank 2007), which are projected to be more frequent because of climate change in Swaziland (Manyatsi, Mhazo & Masarirambi 2010).

A critical factor that influences the adaptive capacity of communities is their access to and control over natural, human, social, physical and financial resources (Simane et al. 2012). Access to and control over the resources necessary for adaptation is influenced by many other factors such as policies, institutions and power structures (Dulal, Brodnig & Shah 2010). For climate change adaptation to be effective, it is important that the enabling environment offers the poor population like SSFAs of Swaziland the rights, resources and access they require to sustain and benefit from ecosystems, public services and markets. This is because the poor are often faced with lack of resource rights and inadequate access to markets, finance, information and technology, resulting in increased vulnerability compared to climate change effects (WRI 2009). Smallholder adaptive capacity to climate change risks is generally lowered by overdependence on natural resources, limitations in human and physical capital as well as poor infrastructure (Shewmake 2008). Therefore, in this study, the adaptive capacity of the SSFAs was explored in order to determine and identify what adaptive strategies may already be operational and what may need to be designed and/or supported in response to future climate variability and change. The next section draws attention to materials and methods used in generating and analysing data.

## Materials and methods

**FIGURE 1:**
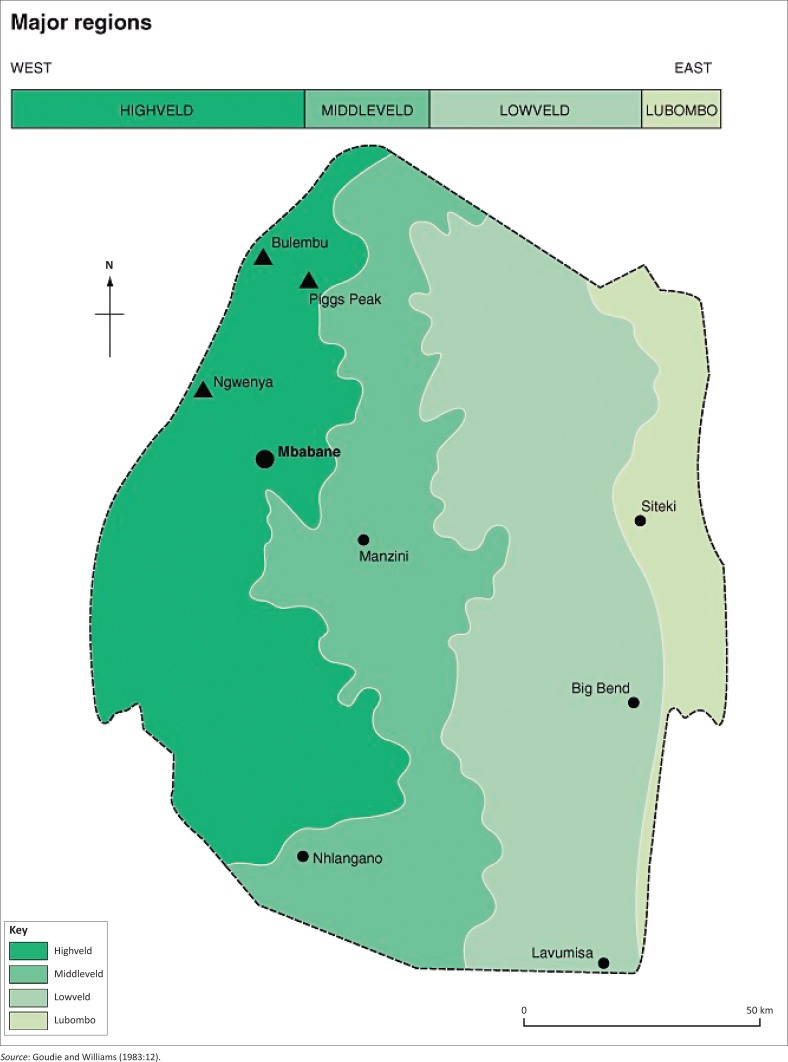
Location of study area.

The Lowveld region is the study site (Figure 1). The main reason for selecting it is that it has about 70% of the 400 sugarcane farmers in Swaziland. In addition, the literature reveals that climate variability and change will be more severe in this region (Matondo et al. 2004). An estimated 45 SSFAs made up the total population for the study, and these came from the Komati Downstream Development Project (KDDP), Royal Sugar Corporation (RSSC) and Outgrowers as well as Ubombo and Outgrowers of the Lowveld.

On average, an SSFA is made up of 60 individual farmers who amalgamate their small portions of land. On the technical front, the SSFAs have supervisors who are part of the committees that run the SSFAs. Given the knowledge and hands-on approach of the supervisors, the work purposively sampled these supervisors with 10+ years’ stay on the farms as respondents to the survey questionnaire. To this end, a total of 45 supervisors (representing 45 SSFAs and an estimated more than 2700 farmers) was the realised sample. Further details on the population and sample sizes are indicated in Table 1. The literature (SSA 2011) shows that small-scale farmers usually own land amounting to about one ha individually, which they pull together into the SSFAs. The smallest SSFA encountered was about 68 ha, while the largest SSFA encountered was 305 ha.

**TABLE 1:** Small-Scale Famers Associations in the study area (*n* = 45).

Location in the Lowveld	SSFAs (1–4 years old)	SSFAs (5–9 years)	SSFAs (10+ years)	Sample
KDDP small-scale	5	18	14	14
RSSC + outgrowers	-	36	16	16
Ubombo + outgrowers	-	106	15	15
**Total sample**				**45**

SSFAs, Small-Scale Famers Associations.

The questionnaire contained multiple-choice, closed-ended questions that were for quantitative analysis and open-ended questions for qualitative analysis. Data for closed-ended questions were entered into the Statistical Package for Social Sciences (SPSS) software programme for analysis and plotting of descriptive statistics. On the other hand, the inclusion of open-ended questions was critical to allow the respondents to think ‘outside the box’ letting them use their own words to narrate their experiences and also aiding in giving detailed account of events from further probing by interviewers. Research assistants administered the questionnaires in person and this meant that all responses on the instrument were filled in. There were no instances of respondents indicating they were not comfortable with any questions as this was cleared during the pilot phase. There was also an opportunity given for the respondent to give additional comments or make any other contributions on issues that may not have been included in the questionnaire.

Sample questions included one like, ‘please rate which of the following operations are influenced by too much rainfall’, with ploughing time, planting time, weeding, irrigation, fertiliser application, repening, burning, cutting and transport/haulage as options. The same closed responses were utilised for probing questions on ‘too little rainfall’, ‘too much heat’ and ‘too low heat’. Other questions asked for types of fertilisers used, time of the day at which irrigation took place and sources of climate change information with options for radio, extension officers, newspaper and ‘other’. Questions on adaptive capacity were further asked, such as, ‘which of the adjustments listed below did you do or plan to do to address impacts of climate change in order of importance?’ The responses were mainly closed-ended, and included change crop variety, change planting time, build a water reservoir, implement soil conservation techniques, get an insurance policy, increase irrigation, decrease irrigation, change irrigation system, change irrigations times, change from crop type and other. Some qualitative questions were also utilised to probe responses from key informants. One such question was ‘what are the major challenges experienced and potential areas of improvement in combating climate change in the Lowveld?’ Some of the responses that emerged included lack of information available on the subject, a call for more research and awareness raising, a need for more training and capacity building, and a call for improvement in climate adaptation modelling.

## Presentation of results and discussion of findings

This section presents the result and discussions on adaptive capacity assessment performed following the SLF. Each of the highlighted SLF assets discussed earlier will now be considered in turn in the next subsections.

### Adaptation through natural assets

The natural resource stock upon which farmers obtain resources useful for sugarcane production include land, water, clean air, forests, erosion protection and biodiversity, which the communities utilise for a livelihood. Land is one of the key natural assets available to SSFAs in the study area. The SSFAs have access to land through chiefs and have no individual ownership titles. The results indicate that SSFAs are allocated small pieces of land by the chief and have to pull the land together with other farmers in order to farm profitably. It emerged that the SSFAs tend to mismanage their land, thereby minimising their adaptive capacity to climate variability and change. To this end, if adaptive capacity to climate change impacts of the SSFAs is to improve, land access must be accompanied by land rights enforced through a formal Land Policy. However, the Land Policy has remained in the draft form for a long period in Swaziland.

The study revealed that in an effort to help SSFAs improve land and resource management, the Swaziland Water and Agricultural Development Enterprise (SWADE) has been assisting communities to develop a *Chiefdom* Development Plan (CDP). This CDP guides all development interventions and helps in the identification and transformation of available natural resources into products required for sustainable livelihoods, including addressing the negative impacts of climate change. The CDP development strategies and interventions include livestock commercialisation, environment management, public health, potable water and sanitation, road and electricity infrastructure, community tourism development, land tenure security and other cross-cutting issues such as education, gender equality and access to social grants. In analysing these strategies, the study found out that these interventions are actually very important in promoting adaptive capacities of the SSFAs in the study area. It emerged that SWADE was working in partnership with the Global Environment Facility (GEF) to develop and implement sustainable land management approaches too. The two organisations collaborate to address the root causes of biodiversity loss and the implications for climate change. The project establishes conservation areas and promotes the use of fuel-efficient stoves and solar power for cooking and lighting in households. This initiative helps to improve the adaptive capacity of the SSFAs.

The study revealed that water availability and accessibility is another vital natural resource important for adaptation utilised by SSFAs. The construction of the Maguga and Lubovane dams allowed these farmers access to water for irrigation. Water from the dams ensures a constant supply even during times of drought. Water is also used for watering gardens around farmers’ homesteads and sugarcane farms, an element that stands out as an adaptation measure. The study further revealed that the SWADE, working with other national organisations, has installed water supplies to some communities for other income-generating businesses to help the small-scale farmers improve their livelihoods as an adaptation measure.

**FIGURE 2:**
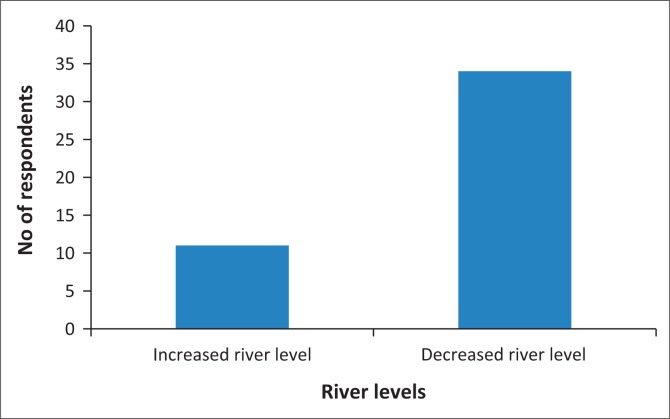
Responses on observed river levels.

Floods and droughts affect sugarcane production in many ways, such as directly impacting on agronomic processes and activities, affecting river water quantity and quality for irrigation purposes, and affecting the functioning of the irrigation system. All these effects in turn affect the yield and quality of the sugarcane crop and the net revenue. Water storage infrastructure is very important for drought conditions, yet the results indicate that 89% of the farmers surveyed stated that they do not have on-farm water storage infrastructure to store irrigation water for use during dry spells. The remaining 11% of the farmers claimed that the storage infrastructure they have is not enough to store water for use during drought conditions. The choice of the right irrigation system for the right climatic conditions and soils is, therefore, very important in sugarcane production as it ensures water use efficiencies and a resultant increase in yield. Farmers have observed drastic modifications in river flow (and by implication the quality, quantity, erosion rate and sedimentation) in the two river basins as confirmed by the results in Figure 2.

Most of the SSFAs in the study area utilise water from the Komati and Usutu River systems with a few utilising water from the Mbuluzi River. However, the farmers stated that the dams have both negative and positive impacts on their livelihoods. The positive impacts include water availability for sugarcane irrigation throughout the year. Negative impacts affecting livelihoods include the fact that flood peaks have increased downstream of the dams, drowning irrigation equipment such as pumps. Silt deposition also worsened over the years as the river flow lessened causing clogging of pipes, thereby reducing the amount of water reaching the sugarcane crop and increasing operation and maintenance costs.

### Adaptation through financial assets

**FIGURE 3:**
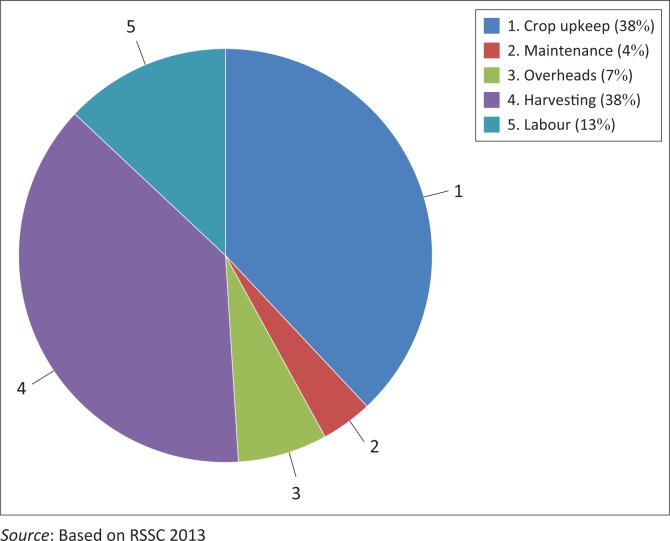
Farm operational cost structure.

After natural assets, financial capital was identified by farmers as one of the most important assets for building adaptive capacity. The study indicates that sugarcane farmers have limited access to credit from banking institutions provided through government support. The main challenge on utilising the financial assets for adaptation, however, is that the loans attract high interest rates and thereby add to the high operational costs that affect net income. The SSFAs’ operation cost structure is reflected in Figure 3. Harvesting costs are significantly higher for the small-scale farmers because of the distance to the mill. The main operational costs incurred in the production of sugarcane comprise harvesting and crop upkeep.

When respondents were asked about the constraints that hinder them from employing adaptation measures, 88% cited lack of money, while 12% cited lack of information. The results further indicate that the sugar industry is of high importance to the national government and, therefore, receives attention locally and from international, developmental and donor partners. The government has been in negotiations with the European Union to offer subsidy support to the small-scale farmers, which has come as preferential pricing. The government support has been realised in the support rendered in the expansion of irrigation activities through SWADE and the Lower Usutu Smallholder Irrigation Project (LUSIP). Judging from the amount of support the sugar industry is currently receiving from the government, prospects are high that adaptive capacity through financial assets is set to improve in the study area despite the economic turmoil faced.

### Adaptation through physical assets

The study shows that generally the availability of physical assets is not a major challenge as the SSFAs are able to access tractors and other farm equipment. The use of computers, telephone, email and cellophane for communication and improved farm management is very poor within SSFAs. An estimated 86% of these farmers rely on agriculture extension staff for information dissemination on both agriculture and climate. All respondents rated the cellular phone (100%) as the most widely used means for information exchange, including in times of emergency. This was followed by the radio (Walkman), which was rated second by 40% and notice boards. Nevertheless, cellular phone usage depends on network coverage for effective communication, an aspect which is still very poor in the study area. The most common sources of climate information among farmers are radio and television.

Additional revelations were that only 3% of the respondents had a drought strategy. The 97% of the respondents who do not have this strategy indicate that the farmers are not prepared for climate change, and thus, their adaptive capacity is quite low for drought-related stresses, yet it is so prevalent. All the respondents indicated that they have no early warning systems or flood management strategy for the climatic stresses already experienced and forecasted. When the respondents were asked what adaptive measure they would implement first against climate change, building a reservoir was selected as the most important measure, followed by increasing the extent of irrigation. This is particularly a true reflection because 89% of the respondents indicated that they do not have water storage infrastructure, and of the 11% storage infrastructure, the response was that it is not enough to cater for water shortages during drought incidences when water requirement for the crop is high. Other adaptive measures that emerged included changing from one crop to another crop, changing planting and irrigation times, changing irrigation systems, changing crop variety and acquiring insurance.

### Adaptation through human assets

Sugarcane farming is a labour-intensive industry, and therefore, the availability of the human asset is very important. The farmers in the study area have access to informal and formal education systems provided by the national government through the University of Swaziland and the National Agriculture Skills Training College, and through capacity-building courses and demonstration training offered by SWADE, SSA and Ubombo extension services. Swaziland Water and Agricultural Development Enterprise has helped a great deal in capacitating SSFAs through their community empowerment process, which culminates in the development of businesses that serve as a vehicle for wealth creation. The SSA, RSSC and Ubombo extension services also offer mentoring services not covered by SWADE. The training received by the farmers has improved adaptation in the study area. Each SSFA gets support from an extension officer from SSA, SWADE and RSSC. However, it was revealed during the study that education and human capital endowments increase the likelihood of embracing new technologies, as they enhance the ability of farmers to perceive climate change. However, farmers from the SSFAs were not informed, skilled or trained. This situation reduces their adaptive capacity.

### Adaptation through social assets

The farmers in this study area have access to a number of social assets that are enabled mainly through formal and informal institutions. Extension services from SSA, RSSC, SWADE and Ubombo Sugar are available and are offered through field visits, seminars, capacity-building and training sessions. The SSA has developed training manuals that cover all the resources needed to grow sugarcane (e.g. climate, soils, water, labour, transport, equipment and capital) as well as all the technical aspects of production (e.g. varieties; land preparation; weeds, disease and insect control; irrigation; fertiliser recommendations; chemical ripening; harvesting and cane quality) with details of choices available. On many occasions, in collaboration with extension officers, farm input and equipment suppliers offer advisory services to farmers through workshops and meetings. Farmer groups, associations and mill boards are active in this area. Farm visits and networking days are organised through extension officers. The established networks and institutions encourage participation in decision-making, empower farmers and promote knowledge sharing, which has helped farmers to adapt. The working relationship between large- and small-scale farmers also helps to improve adaptive capacity in the sugar industry. It is important to also mentor small-scale farmers on the climate change issues such that it is mainstreamed into daily operations. Key informants revealed that capacity building remains the key and that investment in technological measures is also crucial for improved adaptation. Selected responses from the key informants on their role, the main challenges on combating climate change and proposed ways to sustain adaptive capacities within the sugar industry and the country are listed in Table 2.

**TABLE 2:** Adaptive measures for climate change.

Key informants	Role of institution in addressing climatic disasters before and after	Major challenges and areas of improvement in combating climate change in the Lowveld	Sustained climate change adaptive capacity
Ministry of Agriculture	Policy formulation towards climate change adaptation Programmes that encourage climate change Capacity building Awareness raising on predicted climate change disasters to farmers Promoting crops that are climate resilient Information dissemination that is user-friendly for farmers	Not enough information available on the subject More research needed Awareness raising on climate change More training and capacity building Technological improvement for modelling	Formulate and implement policies
United Nations Development Programme	Support government on climate proofing agriculture through fast tracking policies and implementing programmes	Not enough information available on the subject More research needed More training and capacity building	Formulate and implement climate change responsive policies Invest in climate change programmes
Ministry of Natural Resources and Energy	Policy formulation on climate adaptation related to natural resources, especially water resources	Rainfall prediction is a challenge Agriculture consumes about 96% of the water resources in Swaziland	Investments on technological improvement
Ministry of Economic Planning and Development	Ensuring that sectors include policy formulation on climate adaptation in planning Supporting government in prioritisation of economic growth sectors	It is not yet clear what climate change is and how climate change will affect the economy	Clear communication on impacts and necessary adaptation measures is needed
Meteorology Department	Data collection and storage on weather Inform policy formulation on climate change Information dissemination on weather and related disasters Early warning	Not enough data exist for prediction at a smaller scale A few gauging statins exist and even the few that are there, not all weather stations are functional	Invest in data collection, management and monitoring
Swaziland Water and Agricultural Development Enterprise	Capacity building Awareness raising on predicted climate change disasters to farmers	Not precise information on climate change Early warning systems not available Sugarcane grows over several years and, therefore, makes it difficult to implement quick adaptation measures	Invest in capacity building and awareness
Royal Sugar Corporation	Capacity building Awareness raising on predicted climate change disasters to farmers	Not precise information on climate change Early warning systems not available Sugarcane grows over several years and, therefore, makes it difficult to implement quick adaptation measures	Invest in capacity building and awareness
Ubombo	Capacity building Awareness raising on predicted climate change disasters to farmers	Not precise information on climate change Early warning systems not available Sugarcane grows over several years and, therefore, makes it difficult to implement quick adaptation measures	Invest in capacity building and awareness
Swaziland Sugar Association	Capacity building Awareness raising on predicted climate change disasters to farmers Facilitating early warning mitigation measures with the Swaziland Cane Growers Association, among others	Not precise information on climate change Early warning systems not available Sugarcane grows over several years and, therefore, makes it difficult to implement quick adaptation measures	Invest in capacity building and awareness

The study revealed that the sugar industry is highly regulated through institutional structures and regulations. The main players involved in policy formulation towards the sugar industry include the Ministry of Agriculture, Ministry of Economic Planning and Development and Ministry of Natural Resources and Energy. The Meteorology Department is pivotal for climate data and disaster warnings. The United Nations Development Programme (UNDP) has contributed greatly in capacity-building initiatives at both national and local level. The UNDP has contributed through financial and technological means towards the establishment of institutions to fast-track the policy formulation process. The *Sugar Act* provides for the operation of the SSA and an agreement, which regulates the affairs of the sugar industry. The regulated setup within the sugar industry ensures reliable income generation through improved cooperation and the agreement on sugar sales. Adaptive capacity of the farmers is promoted in that the *Sugar Act* covers the sugarcane production, administration and coordination of sugarcane supply to the mills, and establishes dispute resolution structures, the pooling of the proceeds from the sale of sugar and molasses and the sharing of the net proceeds between the growers and milling sections. The agreement further covers the calculation of the price paid for cane deliveries and cost sharing aspects.

Other Acts and policies that guide sugarcane production include the *Water Act, Environment Management Act*, the Draft Land Policy and Irrigation Policy. *The Environment Management Act* promotes sustainable, efficient and equitable use of natural resources that support sugarcane production. Awareness on environmental degradation by large-scale agriculture such as sugarcane production has resulted in strict requirement for Environmental Impact Assessments and Comprehensive Mitigation Plans. The promulgation of the *Water Act* in 2003 tightened the water permitting system, which changed the allocation system from a volumetric system to a crop water requirement system.

## Conclusions and recommendations

The objective of this study was to assess the adaptive capacities of SSFAs in Swaziland’s Lowveld to climate variability and change. Although the methodology did not set out to calculate an adaptive capacity index, the results point to SSFAs that have difficulty in adapting to climate change. Key factors highlighted include the lack of appropriate and adequate information, lack of research from extension services, lack of awareness and appropriate technology, lack of capacity and inadequate early warning systems. Overall, SSFAs do not have enough flexibility, decision-making and control over their natural and capital assets, particularly land and access to credit, in order to effect changes in production, irrigation and management systems as and when required. Close to 90% of the respondents indicated that they do not have water storage infrastructure. The analysis revealed that SSFAs suffer from external parameters with a bearing on their financial assets. Such parameters include the cost of input commodities, sugar prices, inflation rates, taxes and exchange rates. An analysis on accessibility and availability of the social assets indicated that networks exist in the study area that promote working together, skills development, cooperation and cost sharing. Human assets analysis indicated that there is a skills challenge faced by the SSFAs, worsened by migration of skilled labour to better paying jobs in urban areas and through employment by large-scale farms. Overall, the findings point out that the SSFAs are undertaking a number of coping and adaptation measures.

To this end, the study recommends that if SSFAs are to improve their adaptive capacity through natural and capital assets, then the Government of Swaziland must enact the much awaited land policy to guide the utilisation and preservation of land resources, especially in rural areas. In addition, farmers should move to sugarcane varieties, which are high yielding, drought tolerant, more resistant to pests and diseases and have shorter periods of maturity, which require substantial capital outlays. Therefore, improving financial flows through reduced interest rates on loans and granting land title deeds would contribute greatly in providing buffers as the SSFAs adapt to the changing climate. The government is further encouraged to review credit conditions for SSFAs, developing weather-based index insurance schemes and review tax and charges for fuel and electricity as well as providing subsidies. Other measures to consider include improved provision and dissemination of relevant climate information, which must include climate-smart agriculture and early warning mechanisms. Lastly, the government and other players need to build water storage infrastructure.
